# Mechanisms of GLP-1 in Modulating Craving and Addiction: Neurobiological and Translational Insights

**DOI:** 10.3390/medsci13030136

**Published:** 2025-08-15

**Authors:** Gabriel Amorim Moreira Alves, Masatoki Teranishi, Ana Claudia Teixeira de Castro Gonçalves Ortega, Frank James, Arosh S. Perera Molligoda Arachchige

**Affiliations:** 1 Faculty of Medicine, Humanitas University, Pieve Emanuele, 20072 Milan, Italy; 2 Faculty of Medicine and Surgery, University of Milan, 20122 Milan, Italy; 3 Public Health Department, Lummi Nation Tribal Health Center, Bellingham, WA 98226, USA; 4 Emergency Service, GHOL Hôpital de Nyon, 1260 Nyon, Switzerland

**Keywords:** GLP-1 receptor agonist, substance use disorder, craving, reward circuitry, gut–brain axis, mesolimbic pathway, dopamine, synaptic plasticity, vagal signaling, neuroinflammation, semaglutide, addiction

## Abstract

Substance use disorders (SUDs) remain a major public health challenge, with existing pharmacotherapies offering limited long-term efficacy. Traditional treatments focus on dopaminergic systems but often overlook the complex interplay between metabolic signals, neuroplasticity, and conditioned behaviors that perpetuate addiction. Glucagon-like peptide-1 receptor agonists (GLP-1RAs), originally developed for type 2 diabetes and obesity, have recently emerged as promising modulators of reward-related brain circuits. This review synthesizes current evidence on the role of glucagon-like peptide-1 (GLP-1) and its receptor in modulating craving and substance-seeking behaviors. We highlight how GLP-1 receptors are expressed in addiction-relevant brain regions, including the ventral tegmental area (VTA), nucleus accumbens (NAc), and prefrontal cortex (PFC), where their activation influences dopaminergic, glutamatergic, and GABAergic neurotransmission. In addition, we explore how GLP-1 signaling affects reward processing through gut–brain vagal pathways, hormonal crosstalk, and neuroinflammatory mechanisms. Preclinical studies demonstrate that GLP-1RAs attenuate intake and relapse-like behavior across a range of substances, including alcohol, nicotine, and cocaine. Early-phase clinical trials support their safety and suggest potential efficacy in reducing craving. By integrating findings from molecular signaling, neurocircuitry, and behavioral models, this review provides a translational perspective on GLP-1RAs as an emerging treatment strategy in addiction medicine. We propose that targeting gut–brain metabolic signaling could provide a novel framework for understanding and treating SUDs.

## 1. Introduction

Addiction is a chronic, relapsing neuropsychiatric disorder characterized by compulsive substance use despite adverse consequences, high rates of relapse, and a growing global burden of disease [1]. Conventional pharmacological treatments, such as opioid antagonists, dopamine (DA) reuptake inhibitors, and nicotinic partial agonists, target specific neurotransmitter systems involved in reinforcement and craving. However, these therapies often yield only modest long-term benefits, highlighting the need for novel strategies that address the complex interplay of neurobiological, behavioral, and metabolic drivers of addiction [2].

Emerging evidence suggests that metabolic signaling pathways, long considered peripheral to brain reward systems, may directly modulate central mechanisms underlying substance use disorders (SUDs). In particular, the gut–brain axis has garnered attention as a bidirectional system capable of regulating mood, motivation, and reward-seeking behavior via endocrine, neural, and immune pathways [3,4]. Within this framework, glucagon-like peptide-1 receptor agonists (GLP-1RAs), initially developed for the treatment of type 2 diabetes and obesity, have been identified as promising candidates for modulating the neural circuits involved in addiction [5,6,7].

Glucagon-like peptide-1 (GLP-1) is a gut-derived incretin hormone secreted by enteroendocrine L-cells in response to nutrient ingestion, with additional production by preproglucagon neurons in the Nucleus Tractus Solitarius (NTS). Its receptor, glucagon-like peptide-1 receptor (GLP-1R), is expressed not only in metabolic centers like the hypothalamus but also in reward-related brain regions, including the ventral tegmental area (VTA), nucleus accumbens (NAc), and prefrontal cortex (PFC) [8,9,10]. This distribution enables GLP-1 to influence a range of processes relevant to addiction, including dopaminergic neurotransmission, synaptic plasticity, and the encoding of reward cues. In the remainder of this review, we refer to endogenous GLP-1 simply as ‘GLP-1,’ whereas exogenous GLP-1 is referred to as ‘GLP-1RAs.’

Preclinical studies have demonstrated that GLP-1R activation can reduce the reinforcing properties of addictive substances, suppress drug-seeking behaviors, and attenuate cue-induced reinstatement of use [7,10,11]. Mechanistically, GLP-1R signaling interacts with glutamatergic and gamma-aminobutyric acid (GABA)ergic pathways in mesocorticolimbic circuits, as well as with vagal afferents and hypothalamic networks that integrate energy balance and motivational states [8,12].

Moreover, human data, though limited, suggest that GLP-1RAs may reduce craving and consumption in individuals with alcohol or food addiction, particularly those with coexisting obesity or metabolic dysregulation [13]. These effects, however, must be weighed against the potential for central side effects, including nausea, apathy, or mood disturbances, especially with compounds that cross the blood–brain barrier (BBB) more readily, such as semaglutide.

This review aims to clarify how GLP-1 receptor signaling modulates neurocircuitry involved in SUDs, with a particular focus on the mesocorticolimbic system, gut–brain communication, and synaptic plasticity. We integrate both preclinical and clinical findings to explore the therapeutic potential and mechanistic underpinnings of GLP-1RAs in addiction. While previous reviews have broadly addressed the neuropsychiatric effects of GLP-1RAs, including roles in mood regulation and cognition, our approach centers specifically on addiction-related neurocircuitry, from gut peptides to cortical modulation [14,15].

## 2. GLP-1 and Its Receptor: From Gut Peptide to Central Nervous System (CNS) Signaling

GLP-1 is an incretin hormone produced primarily in the intestinal L-cells and, to a lesser extent, by preproglucagon-expressing neurons in the NTS of the brainstem [3,9]. Peripherally, GLP-1 is secreted in response to nutrient ingestion and regulates insulin release, gastric emptying, and satiety. Centrally, GLP-1 functions as a neuromodulator, with its receptor widely distributed in the brain, including the hypothalamus, NAc, ventral VTA, area postrema (AP), and potentially PFC [5,16] (refer to Table 1). As a neuromodulator, it is capable of modulating both pre- and postsynaptic pathways, affecting glutamatergic and GABAergic neurotransmission [17].

**Figure 1:**
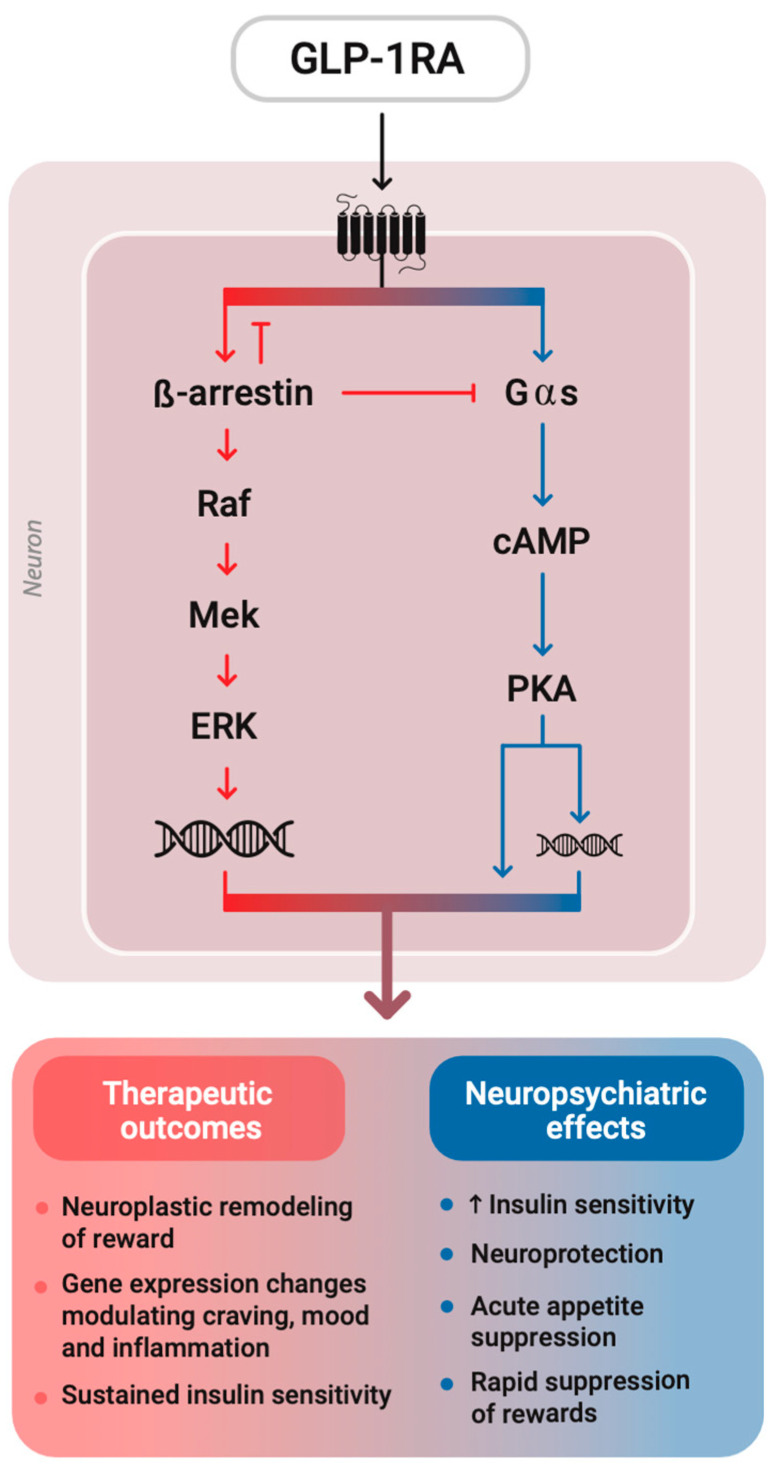
Biased agonism at the GLP-1 receptor and spectrum of downstream signaling. GLP-1RAs can differentially recruit β-arrestin-dependent and Gαs-cAMP-PKA-dependent pathways, producing distinct transcriptional programs within neurons. Rather than acting as binary switches, these pathways exist on a continuum of activation, with specific GLP-1RAs favoring one signaling arm over the other. β-arrestin not only turns off GPCR signaling by blocking G proteins but also steers the receptor to activate gene transcription and support long-term cellular changes. This agonist bias can shape the balance between neuroplastic, metabolic, and behavioral effects, offering opportunities for pathway-selective drug design.

GLP-1R belongs to the class B family of G protein-coupled receptors (GPCRs), signaling primarily through Gαs to increase cAMP levels, which in turn activate protein kinase A (PKA). It also engages other intracellular pathways, including β-arrestin-mediated MAPK signaling, where β-arrestin desensitizes GPCRs by blocking G protein coupling and acts as a scaffold to activate mitogen-activated protein kinases (like ERK), promoting longer-term effects such as gene expression and cell survival by regulating gene transcription, independent of G protein signaling [22]. Recent structural studies reveal that ligand-specific conformations of class B GPCRs modulate β-arrestin recruitment, receptor trafficking, and signaling duration, thereby influencing both therapeutic outcomes and side effect profiles [27]. This structural basis underlies the ligand-specific signaling bias, without being an either–or selectivity. In other words, different GLP-1RAs induce conformational changes to the corresponding GPCR, which shift the balance of signaling towards either a Gαs cascade or a β-arrestin recruitment to varying extents [28]. Semaglutide, for instance, exhibits G protein-biased agonism, favoring prolonged cAMP signaling while limiting β-arrestin recruitment, potentially reducing receptor desensitization and enhancing therapeutic efficacy [17,29]. However, this comes at the potential cost of diminished β-arrestin-dependent pathways (like MAPK signaling), and whether that tradeoff is entirely beneficial remains context-dependent and still under investigation (Figure 1).

A range of GLP-1RAs has been formulated, each exhibiting distinct pharmacokinetic characteristics. For example, liraglutide, a long-acting analog, displays limited CNS penetrance and is associated with fewer neuropsychiatric side effects, while semaglutide exhibits broader central distribution [19,30]. Structural studies have demonstrated that these agents differ in their capacity to induce receptor internalization, duration of signaling, and tissue specificity factors that influence both metabolic and neurobehavioral outcomes [27,29]. Translationally speaking, this signifies that CNS-selective targeting, or avoidance, coupled with the signaling profile, could help tailor GLP-1RAs for specific diseases, patient subgroups, or tolerability profiles.

Although GLP-1 is rapidly degraded peripherally by dipeptidyl peptidase-4 (DPP-4), its central effects are mediated either by DPP-4-resistant analogs or through direct neural projections from GLP-1-producing brainstem neurons. These neurons integrate peripheral metabolic cues via vagal afferents and project directly to the VTA and NAc, where GLP-1R activation decreases food intake and body weight, particularly suppressing consumption of highly palatable foods [12,21]. Importantly, GLP-1R activation in CNS regions associated with motivation and reinforcement suggests a mechanistic bridge between gut-derived metabolic signals and neuropsychiatric regulation, a foundational concept for understanding its emerging role in substance use disorders.

## 3. GLP-1R in Mesocorticolimbic Reward and Relapse Circuits

The mesocorticolimbic DA system, encompassing the VTA, NAc, and PFC, plays a central role in mediating reward, reinforcement learning, and compulsive drug-seeking. GLP-1Rs are expressed across this circuitry, where their activation modulates dopaminergic, glutamatergic, and GABAergic neurotransmission [5,9].

While early studies relied heavily on rodent models to map GLP-1R distribution [9], recent evidence by Gupta et al. provides the first whole-brain mapping of GLP-1R expression in the human brain. Using reverse transcription polymerase chain reaction (RT-PCR) and immunohistochemistry in 30 postmortem brains, GLP-1R expression was confirmed in mesocorticolimbic regions, including the frontal cortex, PFC, hippocampus, and thalamus. These findings underscore the translational relevance of rodent models and highlight potential species-specific differences, particularly in cortical GLP-1R expression relevant to executive function and craving control [25].

GLP-1R-mediated dopaminergic modulation may vary by drug class: in alcohol models, suppression of dopamine release is coupled with reduced locomotor sensitization [7,24], whereas in psychostimulant paradigms, GLP-1R activation primarily blunts drug-induced dopamine overflow without abolishing baseline dopaminergic tone [10]. These nuanced effects indicate that GLP-1RAs are not simply broad-spectrum dopamine blockers; rather, they recalibrate mesolimbic dopamine signaling in a context-dependent manner, preserving baseline dopaminergic function while selectively dampening drug- or reward-driven hyperactivity. For example, in alcohol models, liraglutide attenuates alcohol-induced dopamine release in the nucleus accumbens without affecting normal signaling, and in psychostimulant models, GLP-1RA effects involve modulation of dopamine transporter function rather than global suppression [7,24,31]. This attenuation of phasic dopamine release disrupts the neuroadaptations that underlie craving and relapse, including sensitization of D1 receptor-expressing medium spiny neurons and maladaptive synaptic potentiation in reward circuits.

In the VTA, GLP-1R activation via central administration of GLP-1RAs such as exendin-4 modulates neuronal excitability, in part by enhancing AMPA/kainate receptor activity on glutamatergic inputs and by regulating local GABAergic interneuron tone [10,23]. These effects are partially mediated by enhanced AMPA/kainate receptor activity on glutamatergic inputs and/or modulation of GABAergic interneurons [8,26]. More specifically, exendin-4 increases AMPA/kainate receptor-mediated excitatory postsynaptic currents and reduces paired-pulse ratios in the VTA, indicating enhanced presynaptic glutamate release [5].

Within the NAc core, similar mechanisms have been observed. Mietlicki-Baase et al. demonstrated that exendin-4 selectively enhanced AMPA/kainate receptor activity in medium spiny neurons without altering DA release [32]. The resulting behavioral suppression of high-fat food intake was reversed by the AMPA/kainate antagonist CNQX, confirming a glutamate-dependent pathway. These findings indicate that GLP-1R stimulation modulates mesolimbic excitability through excitatory glutamatergic control rather than direct dopaminergic inhibition.

GLP-1R-mediated modulation of reward circuits extends to the amygdala and medial prefrontal regions. Functional mapping and expression data indicate the presence of GLP-1Rs in the central amygdala (CeA), hippocampus, and PFC, which are implicated in affective modulation of craving and top-down executive control [33]. In the CeA and infralimbic cortex (ILC), systemic semaglutide administration increases spontaneous inhibitory postsynaptic currents (sIPSCs), suggesting enhanced GABAergic tone in regions involved in emotional regulation and extinction learning [26]. Notably, these inhibitory effects were attenuated in alcohol-exposed rats, suggesting that substance use may blunt GLP-1R responsiveness in relapse-relevant circuits. According to Gupta et al., the most abundant expression of GLP-1Rs in humans is found in the frontal cortex, implying that in humans, GLP-1 signaling may engage higher-order cortical mechanisms to regulate eating behavior, extending beyond homeostatic hunger to include executive functions such as impulse control, decision-making, and reward evaluation.

Supporting the neuroanatomical basis for these effects, Alhadeff et al. demonstrated that GLP-1-producing neurons in the NTS project directly to both the VTA and NAc, establishing a physiological bridge through which peripheral metabolic signals influence central reward processing. This highlights the integrated nature of GLP-1 signaling, linking gut-derived hormonal cues to mesocorticolimbic neurotransmission [8].

Furthermore, evidence from psychostimulant models expands the relevance of GLP-1R signaling beyond food reward. Systemic exendin-4 administration attenuates cocaine- and amphetamine-induced locomotor stimulation and conditioned place preference, with concurrent reductions in DA efflux in the NAc [6]. These findings suggest that GLP-1R activation can counteract drug-induced dopaminergic hyperactivity, supporting its therapeutic relevance across SUDs.

Together, these data support a model in which GLP-1 signaling recalibrates mesocorticolimbic excitability and synaptic plasticity via convergent modulation of dopaminergic, glutamatergic, and GABAergic pathways. This multifaceted control may underlie the capacity of GLP-1R agonists to dampen reward salience, reduce craving, and mitigate relapse vulnerability in the context of both natural and drug rewards.

## 4. Gut–Brain Axis and Vagal Modulation of Craving

The gut–brain axis serves as a bidirectional communication system between the gastrointestinal tract and CNS, playing a crucial role in energy homeostasis, reward processing, and motivational states. GLP-1, produced in enteroendocrine L-cells and preproglucagon neurons in the NTS, plays a central coordinating role by integrating peripheral and central signals, then modulating downstream pathways that control appetite, metabolism, and behavior [9,34].

**Figure 2:**
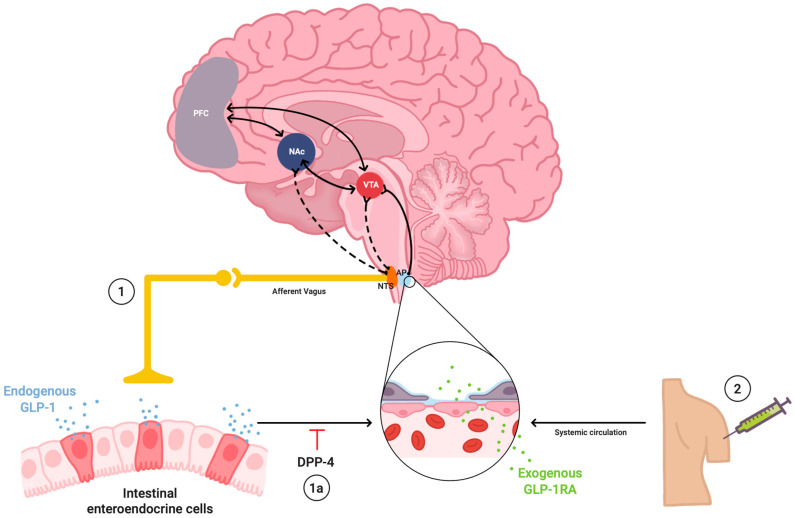
Dual gut–brain pathways for glucagon-like peptide-1 (GLP-1) receptor signaling and behavioral modulation. Nutrient-triggered GLP-1 is secreted from enteroendocrine L-cells in the distal small intestine and colon, reaching the brain via two main routes: (1) activation of vagal afferent fibers that project to the Nucleus Tractus Solitarius, (NTS), and (2) entry into the bloodstream, where it can access central structures such as the area postrema (AP), a circumventricular organ lacking a complete blood–brain barrier (BBB). Some GLP-1s enter the bloodstream through local capillary beds, but inhibition by dipeptidyl peptidase-4 (DPP4) in the vascular endothelium limits their half-life to 1–2 min (1a). Long-acting glucagon-like peptide-1 receptor agonists (GLP-1RAs), such as semaglutide and liraglutide, mimic and amplify the exogenous pathway. Central GLP-1R activation in mesolimbic and mesocortical circuits, including the ventral tegmental area (VTA), nucleus accumbens (NAc), and prefrontal cortex (PFC), modulates dopaminergic, glutamatergic, and GABAergic signaling, thereby reducing reward sensitivity, craving, and substance-seeking behaviors, while also enhancing satiety and impulse control.

Vagal afferents represent the principal route for peripheral GLP-1 influencing brain reward circuits. These sensory fibers innervate the gastrointestinal tract and project to the NTS, where GLP-1-producing neurons are also located [8]. NTS neurons, in turn, send glutamatergic projections to mesolimbic structures such as VTA and NAc, forming an anatomical and functional pathway through which gut-derived signals regulate dopaminergic activity (Figure 2) [16].

In preclinical models, direct activation of GLP-1Rs in the NTS reduces food and drug intake, whereas knockdown of GLP-1R in this region reverses these effects [20,35]. These findings highlight the role of the NTS not only as a relay station but also as a central processing hub for reward suppression. Furthermore, vagotomy or pharmacological blockade of vagal transmission abolishes the effects of peripheral GLP-1RAs on reward behavior, underscoring the importance of intact gut–brain signaling [36]. Altogether, these data support a mechanistic model in which GLP-1R activation modulates craving through gut–brain vagal circuits that converge on reward-related neuroanatomy. This offers a promising mechanism by which GLP-1RAs may exert effects on compulsive intake and relapse behavior in SUDs.

Emerging evidence suggests that vagal GLP-1 signaling capacity is not uniform across individuals and may be influenced by factors such as baseline autonomic tone, metabolic state, and prior substance exposure. For instance, reduced heart rate variability, a surrogate of diminished vagal activity, has been observed in individuals with alcohol use disorder and other addictions, potentially limiting the efficacy of peripherally acting GLP-1 [37]. Moreover, a high-fat diet impairs vagal responsiveness, attenuating the ability of gut-derived GLP-1 to modulate mesolimbic circuits [38]. These interindividual differences highlight the need for incorporating objective measures of vagal function, such as baroreflex sensitivity or heart rate variability, into early-phase clinical trials. Such measures could serve as biomarkers to identify patients most likely to respond to therapies leveraging the gut–brain axis.

From a translational perspective, combining GLP-1RAs with interventions that selectively increase vagal afferent–NTS signaling represents a promising approach. This could include non-invasive neuromodulation techniques (e.g., transcutaneous auricular vagus nerve stimulation), pharmacological enhancement of endogenous GLP-1 release from L-cells, or dietary manipulations designed to optimize postprandial incretin release [39,40]. Trials adopting this multimodal design should integrate stratification by vagal tone and metabolic phenotype, allowing for precision targeting of treatment and minimizing unnecessary drug exposure in low responders. By explicitly operationalizing the vagal pathway as both a therapeutic target and a patient-selection criterion, future research can directly test its contribution to the anti-craving efficacy of GLP-1RAs in substance use disorders.

## 5. Synaptic Mechanisms and Safety Considerations

GLP-1R activation influences synaptic transmission across key nodes of the mesocorticolimbic reward pathway. In the VTA, GLP-1R stimulation enhances AMPA/kainate-type glutamatergic input to dopaminergic neurons, modulating their excitability and reducing the salience of conditioned cues [5]. Similarly, in the NAc, GLP-1R activation increases presynaptic glutamate release and dampens cue-induced reinstatement of drug-seeking, effects that may be mediated by glutamatergic or GABAergic interneuron modulation [24,32].

These changes suggest that GLP-1R signaling exerts its anti-craving effects, at least in part, by altering synaptic plasticity in addiction-related circuits. Synaptic efficacy in the VTA and NAc is central to drug-induced neuroadaptations, and GLP-1RAs may reverse or attenuate these maladaptive changes by modulating excitatory/inhibitory balance [7,31].

However, the pharmacodynamics of GLP-1RAs reveal additional complexity. GLP-1R is a class B GPCR that signals through both Gαs-mediated cAMP/PKA pathways and β-arrestin-mediated MAPK cascades. Some GLP-1RAs, such as semaglutide, display G protein-biased agonism, promoting sustained cAMP signaling while minimizing β-arrestin recruitment [29,41]. This bias may contribute to enhanced metabolic efficacy but also carries implications for receptor desensitization and tissue-specific overstimulation.

In CNS regions associated with affect regulation, such as the amygdala, hypothalamus, and insula, overactivation of GLP-1Rs has been linked to adverse neuropsychiatric effects. Reported side effects include worsening of depression, anxiety, apathy, and, in rare cases suicidality, especially in populations using GLP-1RAs for weight loss without metabolic disease [30,42]. These adverse outcomes may result from prolonged GLP-1R engagement or differential penetration into brain regions, as seen with semaglutide’s high CNS distribution compared to liraglutide in preclinical studies [43]. These points highlight the importance of carefully evaluating a patient’s medical and psychiatric background before initiating GLP-1 RA therapy and point to an urgent need for well-designed prospective clinical studies to clarify the mental health effects of these drugs in individuals with obesity.

Furthermore, internalization kinetics and receptor recycling rates vary by compound, influencing both efficacy and side effect profiles. The balance between central efficacy and tolerability remains a key consideration as dual and triple agonists, targeting GIP and glucagon receptors (GCGRs) in addition to GLP-1, enter clinical use [27,44].

While GLP-1R activation holds promise in reshaping addiction-relevant synaptic activity, careful attention to receptor bias, CNS penetrance, and patient-specific vulnerabilities is essential. Personalized approaches may help optimize neurobehavioral outcomes while minimizing psychiatric risks.

## 6. Hormonal Crosstalk and Metabolic Integration in Reward Modulation

The therapeutic effects of GLP-1RAs extend beyond isolated receptor pathways, engaging broader neuroendocrine networks that regulate energy balance, mood, and motivation. As incretin-based therapies evolve, the emerging class of dual and triple agonists targeting GLP-1, GIP, and GCGRs has shown potential to influence overlapping circuits of metabolism and reward [45].

**Figure 3:**
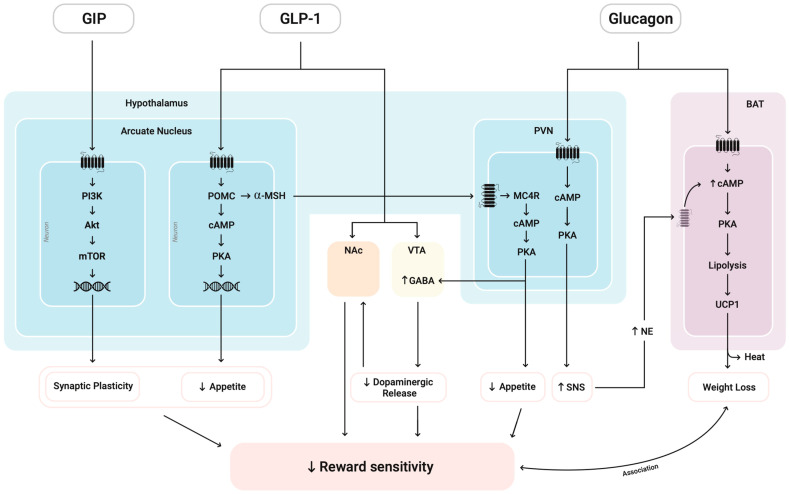
Glucagon-like peptide-1 (GLP-1) reduces appetite via pro-opiomelanocortin (POMC)/cyclic adenosine monophosphate (cAMP)/protein kinase A (PKA) activation and dampens reward signaling through alpha-melanocyte-stimulating hormone (α-MSH)-driven GABAergic inhibition of the paraventricular nucleus (PVN) to ventral tegmental area (VTA), as well as direct modulation of VTA dopaminergic neurons and nucleus accumbens (NAc) glutamatergic tone. Glucose-dependent insulinotropic polypeptide (GIP) promotes phosphoinositide 3-kinase (PI3K)/Ak strain transforming (AKT)/mammalian target of rapamycin (mTOR)-mediated synaptic plasticity, which may enhance top-down control over reward. Glucagon signaling in the PVN increases sympathetic output and norepinephrine (NE), while activation in brown adipose tissue (BAT) promotes thermogenesis via lipolysis and Uncoupling Protein 1 (UCP1). Resulting weight loss and reduced reward sensitivity are interconnected through a bi-directional association that may reinforce healthier behavioral patterns.

As summarized in Figure 3, pro-opiomelanocortin (POMC)-expressing neurons synthesize POMC, which is cleaved into peptides such as α-MSH and released to exert anorexigenic effects [46]. This effect is coupled with a downregulation of dopaminergic tone in mesolimbic structures, including the VTA and NAc, leading to reduced reward sensitivity [13,31]. The paraventricular nucleus (PVN) MC4R pathway also contributes to this by sending glutamatergic projections to the VTA, activating local VTA GABA interneurons [47]. The behavioral consequence of this circuit is well-documented: optogenetic stimulation of VTA GABA neurons directly suppresses the activity and excitability of neighboring DA neurons [48] and, critically, this suppression disrupts reward consumption while leaving anticipatory behavior intact [49]. This established DA suppression pathway initiated by MC4R through PVN represents a direct mechanism by which metabolic signals can modulate reward processing and reduce reward consumption.

GIP receptors are co-expressed with GLP-1Rs in the arcuate nucleus (ARC) and may act synergistically through PI3K/AKT signaling to reinforce anorexigenic output and insulinotropic responses [45]. On the other hand, GCGR activation enhances energy expenditure through mechanisms involving BAT thermogenesis and sympathetic nervous system (SNS) stimulation (Figure 3) [50]. While the SNS’s direct effect on reward sensitivity requires further investigation, the metabolic activation from glucagon may complement the direct reward-suppressing effects of GLP-1 and GIP pathways in SUD treatment.

At the molecular level, crosstalk among intracellular pathways such as cAMP/PKA, AMPK, AKT, and mTOR integrates hormonal signals to calibrate neuronal excitability and synaptic plasticity. By coupling GLP1-R agonist and GIP agonist together (seen in Tirzepatide), reduced reward-seeking behavior induced by the former may be strengthened by the enhanced mTOR effect of the latter, reinforcing neural circuits involved in executive control and decision-making, potentially counteracting the dysregulated reward pathways seen in addiction (Figure 3) [46,51].

The relevance of these mechanisms extends to neuropsychiatric domains. Individuals with obesity and type 2 diabetes, populations commonly treated with GLP-1RAs, show elevated rates of depression, anxiety, and SUDs. Preliminary evidence suggests that incretin-based therapies could be potentially helpful in alleviating these comorbidities by restoring homeostatic signaling in overlapping metabolic and limbic circuits [15,47].

Understanding how hormonal integration shapes behavioral output is particularly important in addiction medicine, where metabolic state, reward drive, and affective regulation are tightly interwoven. The multi-pathway approach could enhance therapeutic efficacy by simultaneously reducing reward sensitivity through direct neural mechanisms (GLP-1/GIP) while providing metabolic benefits that support recovery, potentially making dual- or triple-agonist therapies more effective than single-agent approaches for addressing both the neurobiological and physiological aspects of SUDs. Co-agonists targeting GLP-1, GIP, and/or GCGR hold promise for enhancing metabolic and reward-modulating effects. Yet, increased receptor engagement and potential CNS penetrance may amplify neuropsychiatric risk in susceptible individuals. Careful balancing of polyagonist efficacy against safety, alongside early-phase monitoring for neuropsychiatric outcomes, will be essential in developing these agents for addiction. Further research is needed to delineate how these pathways interact across different receptor targets and patient subtypes.

## 7. Preclinical and Clinical Evidence

A growing body of preclinical research supports the role of GLP-1RAs in modulating reward-related behaviors across a range of addictive substances. Rodent studies have shown that systemic or intracerebral administration of GLP-1RAs, such as exendin-4, liraglutide, and semaglutide, significantly reduces intake, preference, and relapse-related behaviors associated with alcohol, cocaine, nicotine, and highly palatable foods rich in fat and sugar value [5,10,24,31].

In preclinical models of alcohol use disorder (AUD), central GLP-1R activation in reward-related regions such as the VTA and NAc attenuates ethanol intake, conditioned place preference, and stress-induced reinstatement of alcohol-seeking behavior [7]. Jerlhag et. al. further demonstrated that GLP-1RAs suppress alcohol-induced locomotor stimulation and DA release in the NAc, suggesting that GLP-1R signaling can dampen mesolimbic sensitization to ethanol [24].

Clinical studies of GLP-1RAs in alcohol use disorder (AUD) have yielded mixed but promising results. For instance, Klausen et al. conducted a double-blind, placebo-controlled trial showing that exenatide did not significantly reduce heavy drinking days in the overall cohort, but exploratory subgroup analyses revealed a significant reduction in alcohol consumption and craving among participants with obesity (BMI > 30 kg/m^2^). Conversely, lean individuals (BMI < 25 kg/m^2^) showed an unexpected increase in alcohol intake following exenatide treatment [52].

Exenatide was also associated with reduced alcohol cue reactivity in reward-related brain regions and decreased dopamine transporter (DAT) availability [52]. These effects were accompanied by significant weight loss.

In contrast, a Phase 2 clinical trial evaluating low-dose semaglutide demonstrated robust reductions in alcohol consumption during a laboratory-based self-administration task. Participants receiving semaglutide also reported fewer drinks per drinking day and lower weekly alcohol craving scores. Moreover, reductions in heavy drinking over time were predicted with large effect sizes (Cohen’s d > 0.80). Semaglutide also produced clinically meaningful reductions in body weight, suggesting potential dual efficacy in metabolic and substance use outcomes [53].

Similar findings have emerged in nicotine-related studies. Preclinical evidence indicates that GLP-1RAs reduce nicotine self-administration, cue-induced reactivity, and nicotine-induced hyperlocomotion [20,35]. These effects are frequently accompanied by reductions in extracellular DA levels in the NAc, reinforcing the hypothesis that GLP-1R signaling modulates reward processing through dopaminergic pathways. Preliminary clinical studies suggest GLP-1RAs may help attenuate post-cessation weight gain, a common barrier to smoking cessation, although larger randomized trials are ongoing to confirm these findings (NCT05610800).

In the context of psychostimulants, exendin-4 has been shown to blunt cocaine-induced hyperlocomotion, DA overflow, and reinstatement behavior in animal models [10] [26]. The effects appear to rely on GLP-1Rs located in the NAc shell and lateral septum, supporting the notion of region-specific neuromodulation. However, in a clinical study involving individuals with cocaine use disorder (CUD), a single low dose of exenatide failed to reduce craving or euphoria, despite inducing hormonal changes [54]. The limited duration and small sample size may have contributed to the lack of behavioral effects, and ongoing trials are exploring repeated dosing strategies (e.g., NCT06252623, NCT06691243).

Preclinical models of opioid use disorder (OUD) have also demonstrated that GLP-1RAs reduce self-administration and relapse behavior for opioids, including heroin, fentanyl, and oxycodone. These effects involve GLP-1Rs in the NAc shell and central CeA and are likely mediated in part by modulation of stress and anxiety circuits. Moreover, inhibition of DPP-4, which increases GLP-1 levels, has been associated with attenuated opioid withdrawal symptoms and reduced anxiety-like behavior [55,56,57]. Preliminary clinical findings presented at the 2023 AAAS meeting (NCT04199728) reported a 40% reduction in opioid craving with liraglutide, although these results are pending peer-reviewed publication.

In models of compulsive overeating and food addiction, GLP-1RAs consistently decrease binge-like intake of high-fat/high-sugar foods, suppress operant responding for sucrose, and reduce cue-potentiated feeding behaviors [12]. These effects are thought to result from combined modulation of hypothalamic appetite-regulating pathways and mesolimbic reward circuits, as shown in studies using pharmacological antagonists and viral knockdown techniques targeting GLP-1Rs. In clinical contexts, liraglutide has been shown to reduce orbitofrontal and striatal activation in response to food cues and to lower reward-driven eating in observational cohorts. In a randomized trial (NCT01739049), liraglutide significantly improved binge eating scores and body weight among non-diabetic obese individuals, along with beneficial metabolic and cardiovascular effects.

Together, these findings support a compelling translational framework for the use of GLP-1RAs in treating substance and behavioral addictions. However, clinical translation is currently constrained by small sample sizes, heterogeneity in dosing, short trial durations, and an overrepresentation of individuals with comorbid obesity, leaving gaps in evidence for normal-weight patients and those with psychiatric comorbidities. Variability in GLP-1R expression across brain regions, metabolic status, sex, CNS penetrance, and biased agonism among different agents further complicates the interpretation of therapeutic effects. Moreover, while GLP-1RAs show efficacy across multiple addictive substances, the magnitude and nature of these effects likely depend on the neurocircuitry engaged by each drug class. These substance-specific mechanisms, coupled with interindividual variability in GLP-1R distribution and gut–brain signaling capacity, highlight the need for precision-tailored rather than uniform treatment strategies.

The findings presented in this section are summarized in Table 2.

## 8. Discussion

The emerging evidence on GLP-1RAs presents a promising and mechanistically distinct approach to treating SUDs. Originally developed for metabolic regulation, these agents have shown significant potential in modulating central reward pathways by influencing dopaminergic, glutamatergic, and GABAergic transmission across mesocorticolimbic circuits. Preclinical studies consistently demonstrate that GLP-1R activation reduces drug-seeking behaviors, cue-induced reinstatement, and consumption of substances with high addictive potential, including alcohol, nicotine, opioids, stimulants, and palatable food.

The neurobehavioral effects of GLP-1RAs appear to be mediated through a combination of direct central actions and gut–brain signaling via vagal afferents, highlighting the importance of the gut–brain axis in addiction regulation. At the cellular level, GLP-1RAs influence synaptic plasticity and neuronal excitability, potentially reshaping maladaptive neuroadaptations that underlie craving and relapse.

Despite encouraging preclinical and early clinical evidence, several challenges remain. Variability in CNS penetrance, receptor signaling bias, and the potential for neuropsychiatric side effects warrant careful patient selection and compound-specific evaluation. Moreover, human studies remain limited and heterogeneous, emphasizing the need for well-powered, placebo-controlled trials that assess both efficacy and safety across diverse populations and addiction types.

Looking forward, the integration of metabolic and neuropsychiatric care via agents such as GLP-1RAs may reshape how we conceptualize and treat addiction. Further investigation into receptor pharmacodynamics, neural circuit engagement, and hormonal crosstalk, particularly in the context of emerging dual- and triple-agonist therapies, is essential to unlocking their full therapeutic potential. While GLP-1R signaling represents a mechanistically distinct and clinically relevant target in the treatment of addiction, continued exploration of its neurocircuit-level actions, safety profile, and patient-specific responses may open new frontiers in the integration of metabolic and psychiatric care.

Building on the mechanistic overlap between GLP-1RA pharmacology and vagal GLP-1 signaling, future studies should assess the therapeutic potential of combined approaches. Such strategies could include pairing GLP-1RA administration with interventions that selectively enhance vagal afferent–NTS signaling, whether via neuromodulation, gut-targeted pharmacology, or postprandial hormone modulation. This multimodal design may allow for reduced GLP-1RA dosing, mitigating adverse effects while maintaining or even amplifying anti-craving efficacy. Importantly, trial designs should include stratification-based gut–brain signaling capacity, such as vagal tone, and metabolic phenotype, as these factors could determine treatment responsiveness. By integrating these parameters into early-phase trials, researchers may identify patient subgroups most likely to benefit, paving the way for a precision-medicine approach to addiction treatment.

In summary, GLP-1RAs offer a novel strategy for addiction treatment, with the potential to address both the neurobiological and physiological aspects of SUDs. However, additional research is needed to fully understand their mechanisms of action, optimize their use, and ensure their safety and efficacy across different populations and types of addiction.

## Figures and Tables

**Table 1:** GLP-1R activation and its neuromodulatory functions by brain region.

Brain Region	Function	Mechanism	Regeneration/Neuroprotection	Key References
Area Postrema (AP)	Triggers nausea and vomiting	Activates GLP-1Rs on AP neurons outside blood–brain barrier (BBB), stimulates the emetic reflex	No regenerative/neuroprotective role known	[18]
Nucleus Tractus Solitarius (NTS)	Triggers nausea	Especially activates mNTS by crossingthe BBB	No regenerative/neuroprotective role known	[19]
Hypothalamus	↓ Appetite, regulates energy balance	Activates GLP-1R on POMC neurons (↑), inhibits NPY/AgRP (↓), and modulates PVN	May ↓ hypothalamic inflammation and support metabolic set-point recovery	[20]
Ventral Tegmental Area (VTA)	Inhibits drug reward, motivation	↓ Dopamine (DA) neuron firing associated with ↑ VTA GABA neurons	May ↓ neuroinflammation in the context of Parkinson’s	[21,22]
Nucleus Accumbens (NAc)	↓ Food and drug reward	Inhibits DA signaling and alters synaptic plasticity	May ↓ reward hypersensitization after addiction, but direct data is limited	[23,24]
Prefrontal Cortex (PFC)	Associated with spatial cognitive memory	↑ GABAergic tone and sIPSCs in the infralimbic cortex; mixed effects of semaglutide on GABA signaling in ILC	May be used to treat Alzheimer’s	[25,26]

**Table 2:** Summary of preclinical and clinical evidence for GLP-1RAs in modulating substance and behavioral addictions.

Substance/Behavior	Preclinical Findings	Clinical Findings	Key References
Alcohol	↓ Ethanol intake, conditioned place preference, stress-induced reinstatement via VTA and NAc GLP-1R activation; ↓ DA release; and ↓ locomotor stimulation	Exenatide notably decreased heavy drinking days and overall alcohol consumption in a subgroup of obese patients (NCT03232112).Low-dose semaglutide shows initial evidence of reducing craving and some drinking outcomes, warranting larger trials for alcohol use disorder (NCT05520775).	[7,11,31,52,53]
Nicotine	↓ Self-administration, cue-reactivity, hyperlocomotion; ↓ extracellular DA in NAc	GLP-1RAs reduced post-cessation weight gain, potentially supporting smoking cessation. Further randomized control trial (RCT) studies with a larger cohort are needed.Actively recruiting RCT with larger cohort: NCT05610800.	[24,52,58,59,60]
Cocaine	↓ Cocaine-induced hyperlocomotion, DA overflow, and reinstatement behavior via NAc shell and lateral septum GLP-1Rs	No reduction in self-administration, euphoria, or craving after a single low-dose exenatide in CUD patients (RCT). Hormonal changes observed. The study is limited by acute dosing and a small sample. Actively ongoing RCTs such as NCT06252623 and NCT06691243.	[10,54]
Food Addiction/Overeating	↓ Binge-like intake of high-fat/high-sugar foods; ↓ operant responding for sucrose; ↓ cue-potentiated feeding; and modulation of hypothalamic and mesolimbic circuits	Liraglutide reduced orbitofrontal and striatal activation to food cues; semaglutide was associated with reduced reward-driven eating in observational studies.Liraglutide also significantly reduced binge eating scores and body weight in non-diabetic obese individuals, alongside improvements in metabolic and cardiovascular markers (NCT01739049). Actively ongoing RCT: NCT07042672.	[5,13,61]
Opioids	↓ Heroin, fentanyl, and oxycodone self-administration and reinstatement (cue-, drug-, stress-induced) via NAc shell and CeA; DPP-4 inhibition reduces withdrawal and anxiety symptoms	↓ Opioid craving by ~40% with liraglutide in individuals with OUD (preliminary RCT, NCT04199728; AAAS 2023 presentation); results pending peer-reviewed publication.	[55,56,57,62]

## Data Availability

No new data were created or analyzed in this study. Data sharing is not applicable to this article.

## Abbreviations

The following abbreviations are used in this manuscript:

α-MSH	Alpha-Melanocyte-Stimulating Hormone
AMPA	α-Amino-3-Hydroxy-5-Methyl-4-Isoxazolepropionic Acid
AKT	Ak Strain Transforming
AP	Area Postrema
ARC	Arcuate Nucleus
BAT	Brown Adipose Tissue
BBB	Blood–Brain Barrier
cAMP	Cyclic Adenosine Monophosphate
CeA	Central Amygdala
CNS	Central Nervous System
DA	Dopamine
DPP-4	Dipeptidyl Peptidase-4
ERK	Extracellular Signal-Regulated Kinase
GABA	Gamma-Aminobutyric Acid
GCGR	Glucagon Receptor
GIP	Glucose-Dependent Insulinotropic Polypeptide
GIPR	Glucose-Dependent Insulinotropic Polypeptide Receptor
GLP-1	Glucagon-Like Peptide-1
GLP-1RA	Glucagon-Like Peptide-1 Receptor Agonist
GLP-1R	Glucagon-Like Peptide-1 Receptor
GPCR	G Protein-Coupled Receptor
ILC	Infralimbic Cortex
MAPK	Mitogen-Activated Protein Kinase
MC4R	Melanocortin-4 Receptor
mTOR	Mammalian Target of Rapamycin
NAc	Nucleus Accumbens
NE	Norepinephrine
NPY	Neuropeptide Y
NTS	Nucleus Tractus Solitarius
OUD	Opioid Use Disorder
PFC	Prefrontal Cortex
PI3K	Phosphoinositide 3-Kinase
PK/PD	Pharmacokinetics/Pharmacodynamics
PKA	Protein Kinase A
POMC	Pro-opiomelanocortin
PVN	Paraventricular Nucleus
RCT	Randomized Controlled Trial
RT-PCR	Reverse Transcription Polymerase Chain Reaction
sIPSC	Spontaneous Inhibitory Postsynaptic Current
SNS	Sympathetic Nervous System
SUD	Substance Use Disorder
UCP1	Uncoupling Protein 1
VTA	Ventral Tegmental Area
